# Dr. Franklin R. Barross

**Published:** 1896-09

**Authors:** 


DR. FRANKLIN R. BARROSS, of Attica, N. Y., died at his residence in that village, Monday, August 17, 1896, aged 64 years. He had been a prominent physician in active practice for about forty years, and commanded the respect of his acquaintances and neighbors. He will be missed by many personal friends as well as numerous families that he has served in the capacity of physician. His colleagues of the medical profession, with whom he has been so long associated, will also keenly feel his loss. He was a member of the Wyoming county medical society, and for many years had been a United States pension examiner.

Dr. Barross had been afflicted with Bright's disease of the kidneys for some time, but did not relinquish his practice until about three weeks before his death. He went to Clifton Springs, expecting that a rest would be beneficial. This, however, proved disappointing, and he returned to Attica a few days previous to his death.

Dr. Barross, in 1857, married Harriet, daughter of John Sargent, Esq., of Cowlesville, N. Y., who, with one son and two daughters, survives him. His funeral was largely attended.

